# Resistin secreted by porcine alveolar macrophages leads to endothelial cell dysfunction during *Haemophilus parasuis* infection

**DOI:** 10.1080/21505594.2023.2171636

**Published:** 2023-02-08

**Authors:** Kexin Hua, Tingting Li, Yanling He, Aohan Guan, Liying Chen, Yuan Gao, Qianshuan Xu, Haoyu Wang, Rui Luo, Ling Zhao, Hui Jin

**Affiliations:** a State Key Laboratory of Agricultural Microbiology, Huazhong Agricultural University, Wuhan, China; b College of Veterinary Medicine, Huazhong Agricultural University, Wuhan, China; c Hubei Provincial Key Laboratory of Preventive Veterinary Medicine, Huazhong Agricultural University, China; d Department of Animal Disease Diagnosis, Hubei Animal Disease Prevention and Control Centre, Wuhan, China

**Keywords:** *Haemophilus parasuis*, resistin, tight junctions, LKB1/AMPK/mTOR pathway, outer membrane lipoprotein a, exudative inflammation

## Abstract

*Haemophilus parasuis* (*H. parasuis*) causes exudative inflammation, implying endothelial dysfunction during pathogen infection. However, so far, the molecular mechanism of endothelial dysfunction caused by *H. parasuis* has not been clarified. By using the transwell-based cell co-culture system, we demonstrate that knocking out *resistin* in porcine alveolar macrophages (PAMs) dramatically attenuated endothelial monolayer damage caused by *H. parasuis*. The resistin secreted by PAMs inhibited the expression of the tight junction proteins claudin-5 and occludin rather than the adherens junction protein VE-cadherin in co-cultured porcine aortic endothelial cells (PAECs). Furthermore, we demonstrate that resistin regulated claudin-5 and occludin expression and monolayer PAEC permeability in an LKB1/AMPK/mTOR pathway-dependent manner. Additionally, we reveal that the outer membrane lipoprotein gene *lppA* in *H. parasuis* induced resistin expression in PAMs, as deleting *lppA* reduced *resistin* expression in *H. parasuis*-infected PAMs, causing a significant change in LKB1/AMPK/mTOR pathway activity in co-cultured PAECs, thereby restoring tight junction protein levels and endothelial monolayer permeability. Thus, we postulate that the *H. parasuis lppA* gene enhances resistin production in PAMs, disrupting tight junctions in PAECs and causing endothelial barrier dysfunction. These findings elucidate the pathogenic mechanism of exudative inflammation caused by *H. parasuis* for the first time and provide a more profound angle of acute exudative inflammation caused by bacteria.

## Introduction

*Haemophilus parasuis* (*H. parasuis*) is a causative pathogen of Glässer’s disease that causes acute inflammation with massive fibrin exudates in the pleuroperitoneal cavity, resulting in the sudden death of piglets and threatening the global pork industry [1]. Unfortunately, the pathogenesis of Glässer’s disease remains unclear. However, several transcriptome analyses have been performed to investigate the molecular mechanisms underlying the pathogenesis of Glässer’s disease, through which considerable upregulation of the hormone resistin was observed in piglet spleens and alveolar macrophages infected by *H. parasuis* [2,3]. Nevertheless, little is known concerning the effect of resistin in Glässer’s disease. Resistin is a multifunctional peptide hormone that is implicated in obesity-mediated insulin resistance and plays a role in inflammatory modulation [4–6]. A previous study has also established the role of resistin in promoting vascular dysfunction in humans. Additionally, resistin increases circulating monocyte adhesion to endothelial cells in atherogenic processes by inducing intercellular adhesion molecule-1 (ICAM-1) and vascular cell adhesion molecule-1 (VCAM-1) expression in human umbilical vein endothelial cells, which is a critical step in the early stages of atherosclerosis [7]. Furthermore, resistin treatment significantly reduces the mRNA and protein levels of tight junction-related proteins such as occludin and zonula occludens-1 (ZO-1), which mediate tight junction assembly, thereby increasing human coronary artery endothelial cell (HCAEC) monolayer permeability [8]. Therefore, we hypothesised that resistin contributes to piglet vascular endothelium injury during *H. parasuis* infection as it induces typical exudative inflammation, which is primarily caused by endothelial barrier impairment [9]. Vascular endothelial cells are tightly connected through a series of protein-protein interactions, resulting in endothelial barrier formation [10]. Tight and adherens junctions are the major connection modes of plasma membranes. They mechanically link nearby cells and seal the intercellular space to maintain selective barrier function of the endothelium [11,12]. Tight junctions are primarily composed of transmembrane proteins, such as claudins, occludin, and junctional adhesion molecules (JAMs), and are the most robust intercellular junction structures of the endothelial barrier that mediate the selective passage of small-molecule proteins (<1 kDa) [13–15]. Endothelial adherens junctions are constructed between adjacent cells by the extracellular regions of the transmembrane glycoprotein vascular endothelial (VE)-cadherin to form a zipper-like seal [16]. The adhesive activity of transmembrane proteins, claudins, occludin, JAMs, and VE-cadherin, modulates the integrity and permeability of the endothelial barrier.

The present study developed a cell co-culture system based on the transwell technique and investigated the effect of resistin secreted by porcine alveolar macrophages (PAMs) on endothelial monolayer integrity and permeability in porcine aortic endothelial cells (PAECs), especially on modulating tight junction protein expression during *H. parasuis* infection. Our study provides a new perspective on *H. parasuis*-induced typical exudative inflammation and suggests a novel perspective for a comprehensive understanding of Glässer’s disease.

## Materials and methods

### Cell and bacterial cultures

PAECs were separated from fresh porcine aorta using the method described by Carrillo [17] and cultured in M199 (Gibco, Gaithersburg, MD, USA) supplemented with 10% foetal bovine serum (Gibco), 2 mM L-glutamine, and 1% endothelial cell growth supplement (Sciencell, Carlsbad, CA, USA). Experiments involving animal subjects were performed in strict accordance with the Guide for Care and Use of Animals in Research of the People’s Republic of China. The Huazhong Agricultural University Scientific Ethics Committee authorized all the experimental protocols (HZAUSW-2020–0011 and HZAUSW-2022–0010). The PAMs cell line 3D4/21 (CRL-2843) was provided by the American Type Culture Collection and cultured as previously described [18]. The resistin-knockout PAMs cell line was generated using the CRISPR/Cas9 system as previously described [18,19]. Additionally, a western blot analysis was used to detect the resistin gene knockout, as described in further detail below. The *H. parasuis* clinical SH0165 strain and its derivatives were cultured as previously described [18]. Wild-type or resistin knockout PAMs were cultured on the lower compartments of the transwell cell culture system to generate the *in vitro* transwell culture model (pore size of 0.1 μm, Biofilm, China), and the upper compartments were transferred to another well plate to culture PAECs. The upper compartments were then reinserted into the transwell system when PAECs and PAMs were cultured to 80–90% confluence to create a co-culture model. Simultaneously, the *H. parasuis* (100 MOI) was added to the medium of the lower compartments to induce persistent PAM infection, and subsequent analyses were conducted at 0, 6, 12, 24, 48, and 2 h post-infection.

### Bacterial adhesion assay

PAMs were cultured to 90% confluence and incubated with the wild-type, Δ*lppA* mutant, or C-*lppA* strains (100 MOI) for 2 h at 37 °C to allow bacterial adhesion. The PAMs were then washed five times with phosphate-buffered saline and trypsinised with 0.25% trypsin. The cell suspension was diluted to the 10^3^ CFU/mL and plated. Adherent bacteria were counted after 4 h.

### Plasmids and antibodies

The primers 5′-CCGGAATTCATGGATGTGGCGGACCCG-3′ and 5′-ATACTCGAGTCACTGCTGCTTGCAGGCT-3′ were used to amplify the full-length coding sequence (CDS) of porcine Liver kinase B1 (*LKB1*), and the primers 5′-CCGGAATTCATGTCATCATGTGTCTCTAG-3′ and 5′-ATACTCGAGCTACTCCATCGCCTCATC-3′ were used to amplify the CDS of porcine Calcium/calmodulin-dependent protein kinase kinase 2 (*CaMKK2*). The fragments were then cloned into pCAGGS-Flag to generate the eukaryotic expression plasmids pCAGGS-LKB1 and pCAGGS-CaMKK2. The primers 5′-TTCCATATGAAGTCGCTGTGTCCCGTGGATGAA-3′ and 5′-TTGCTCGAGTGGCGTCCTCAGGCGACAGCA-3′ were used to amplify the coding sequence of porcine resistin, and the primers 5′-CGCGGATCCTGTGGTGGTGGTGGTTCAAGT-3′ and 5′-ATTCTCGAGTTTGTCTTTGGTAGCAGTTGCCC-3′ were used to amplify the coding sequence of the *lppA* gene of *H. parasuis*. The fragments were then cloned into pET-28a (+) to generate the prokaryotic expression plasmids pET-Resistin and pET-LppA.Table 1 shows a list of the other plasmids used in this study. Anti-resistin polyclonal antibody (#OARB00163) and polyclonal antibody against junctional adhesion molecule 1 (JAM-1) (ARP61361_P050) were purchased from Aviva Systems Biology (USA). Polyclonal antibodies against claudin-5 (#AF5216), occludin (#DF7504), and JAM-2 (#DF2550) were purchased from Affinity Biosciences (Shanghai, China). Polyclonal antibodies against phospho-LKB1 (Thr 189) (#AP0602) and LKB1 (#A2122) were purchased from Abclonal (China). Monoclonal antibodies against CaMKK2 (#16810), phospho-CaMKK2 (Ser 511) (#12818), AMP-activated protein kinase (AMPK) (#5831), phospho-AMPK (Ser 485) (#2535), mammalian target of rapamycin (mTOR) (#2983), and phospho-mTOR (Ser 2448) (#5536) were purchased from Cell Signaling Technology (Danvers, MA, USA), and monoclonal antibodies against β-actin (#AA128) were purchased from Beyotime (Shanghai, China). The antibody against β-actin was diluted at a ratio of 1/50000, and the remaining antibodies used in this study were diluted at a ratio of 1/1000.

**Table 1.** Plasmids used in this study.

Plasmids	Characteristics	Source
pK18mobsacB	Suicide vector, Kan resistance	ATCC 87,097
pK18-UKD	Kan fragment, upstream and downstream sequences of relative genes in pK18mobsacB, Kan resistance	This study
pSHK3	*E. coli* - *H. parasuis* shuttle vector, Kan resistance	[20]
pSHK3-Gm	Kan is replaced by Gm in pSHK3, Gm resistance	[20]
pSHK3-C-*lppA*	A fragment containing the promoter and complete *lppA* ORF in pSHK3-Gm, Gm resistance	This study
pDG459	SpCas9 with 2A-Puro and a cloning backbone for 2 custom gRNAs	Addgene plasmid #100901

### Western blot analysis

PAECs at 70–80% confluence were stimulated with recombined resistin protein (2.5, 5, or 0 nM) for 2 h or co-cultured with *H. parasuis*-infected PAMs. RIPA Lysis Buffer (P0013B, Beyotime, China), together with a protease inhibitor cocktail (Roche, Mansfield, MA, USA), was used to harvest the total protein content of these cells, and a western blot analysis was subsequently performed as previously described after treating the cells for the indicated time [21].

### Transendothelial electrical resistance (TEER) analysis

To investigated the effects of the AMPK/mTOR pathway on the monolayer permeability of PAECs stimulated with resistin, PAECs were stimulated with the specific AMPK activator Metformin (0 μM, Selleck S5958, USA) or the mTOR inhibitor KU-0063794 (0 μM, Selleck S1226, USA) for 2 h and then treated with resistin (0 nM) for 0, 6, 12, 24, 48, and 2 h. To investigated the effects of LKB1 and CaMKK2 on the monolayer permeability of PAECs stimulated with resistin, plasmids pCAGGS-LKB1 (200ng) or pCAGGS-CaMKK2 (200ng) was transfected into PAECs with Lipo2000 (1μg, Invitrogen, USA), and 4 h after transfection, PAECs were treated with recombined resistin protein (0 nM) for another 2 h. For the TEER analysis of PAECs co-cultured with PAMs, the PAECs were cultured as described in “Cell and bacterial cultures” section. The TEER of monolayer PAECs was measured using an EVOM2 volt-ohm metre (World Precision Instruments, Sarasota, FL, USA) according to the instruction manual.

### Quantitative real-time reverse transcriptase – polymerase chain reaction (qRT-PCR) analysis

The total RNA of PAECs at 2 h post-resistin-stimulation was isolated using TRIzol reagent (Invitrogen, Waltham, MA, USA), and the RNA (1 μg) was reverse-transcribed into cDNA using the iScript cDNA synthesis kit (Bio-Rad, Hercules, CA, USA). cDNA was used for qRT-PCR using SYBR Green Supermix (Bio-Rad), and transcripts were standardized to the mRNA level of porcine glyceraldehyde-3-phosphate dehydrogenase (*GAPDH*). Table 2 shows the primer sequences used in the qRT-PCR analysis.

**Table 2.** Primers used in qRT-PCR.

Primer	Sequences (5’-3’)
Claudin5-F	CTTCCTGGACCACAACAT
Claudin5-R	GTACACCTTGCACTGCATAT
Occludin-F	CTATGCTCGTTATCGTGATG
Occludin-R	CCCATACCACCTCCTATTAA
JAM1-F	AGCCTCGTCTGCTATAACAA
JAM1-R	TGTCTTTCCTGGTCACAGAA
JAM2-F	ATTAGTGGCTCCAGCAGTTC
JAM2-R	AGCTGGATTGCCTTCTTTGT
VE-cadherin-F	ACCACGAGATGTGAAGTTCAA
VE-cadherin-R	GTGATGTTGGCCGTGTTATCG
LKB1-F	GGTGCCGTACTTGGAAGATT
LKB1-R	CACCGTGAAGTCCTGAGTGT
CaMKK2-F	TTGGTGTGAGCAACGAGTTC
CaMKK2-R	TTCCGGGTCTCTGAGAGTGA
GAPDH-F	CCCCAACGTGTCGGTTGT
GAPDH-R	CCTGTTCACCACCTTCTTGA

### Deletion mutant and *lppA complementation strain construction*

Table 3 shows a list of all primers used to generate deletion mutants and the *lppA* complementation strain. Recombinant pK18-UKD plasmids containing upstream and downstream fragments flanking relative genes as well as a kanamycin resistance cassette were constructed according to the method described by Wang et al [20]. Subsequently, plasmid pK18-UKD was introduced into *H. parasuis* strain SH0165 via natural transformation, as previously described by Li et al [22]. In brief, the bacterial cells of *H. parasuis* strain SH0165, suspended in logarithmic phase by M-IV medium, were incubated with plasmid pK18-UKD at 37 °C for 0 min and then incubated with 30% glycerol at 25 °C for 0 min. The bacteria were collected and resuspended in tryptone soy broth (TSB) before plating on a tryptone soya agar (TSA) plate containing 0 mg/L kanamycin. TSA plates were incubated at 37 °C for 8 h, and the transformants were identified via PCR. The entire CDS of the *lppA* gene, together with the promoter region, was amplified and cloned into the *H. parasuis* complemented vector pSHK3-Gm to generate pSHK3-C-*lppA*. The resulting plasmid was introduced into the *ΔlppA* mutant through electroporation (2.5 kV, 5 ms), and the transformants were confirmed using PCR.

**Table 3.** The sequences of primers used in generating deletion mutants and the *lppA* complementation strain.

Primers	Sequences (5’-3’)
P1a (*lppA*-up-F)	CGCGGATCCACCGCTTGTGCAGGTGTCACTGCTCGAACA
P2a (*lppA*-up-R)	TTTATCTTGTGCAATGACCACCACCACAAGCTACTAAGAT
P3a (*lppA*-down-F)	CAGAATTGGTTAATTGGGGGCAACTGCTACCAAAGAC
P4a (*lppA*-down-R)	TAACTGCAGACAAGCGGTTGCCTCTGGAGGTGAGCTATCAC
P1b (*lolB*-up-F)	CGCGGATCCACCGCTTGTATCGACACGAGTTGAAAG
P2b (*lolB*-up-R)	TTTATCTTGTGCAATGGTCTAGGTACTAGTCACTC
P3b (*lolB*-down-F)	CAGAATTGGTTAATTGGCGAGATTATCGCCAATT
P4b (*lolB*-down-R)	TAACTGCAGACAAGCGGTCGATTGGCTTCATTGCTT
P1c (*metQ*-up-F)	CGCGGATCCACCGCTTGTCGTATCGGTAGTGCAACT
P2c (*metQ*-up-R)	TTTATCTTGTGCAATGGTGTTCAAAGGTGCGATG
P3c (*metQ*-down-F)	CAGAATTGGTTAATTGCCACGCCATCTTTGAAGT
P4c (*metQ*-down-R)	TAACTGCAGACAAGCGGTCGCTGAGTTACAAGATGG
P1d (*plpA*-up-F)	CGCGGATCCACCGCTTGTGACTTGCCATACAGTGCA
P2d (*plpA*-up-R)	TTTATCTTGTGCAATGCAGAGTAAGGGATAGCAG
P3d (*plpA*-down-F)	CAGAATTGGTTAATTGTGGGCGTACTAATATGGC
P4d (*plpA*-down-R)	TAACTGCAGACAAGCGGTTACCACTTCTCTTTCGGC
P5 (Kan-F)	CATTGCACAAGATAAAAATATAT
P6 (Kan-R)	CAATTAACCAATTCTGATTAG
P7 (C-*lppA*-F)	CGGGGTACCAGTTAAGGGCAGCTTAAGTTA
P8 (C-*lppA*-R)	TATGAGCTCTTTGTCTTTGGTAGCAGTTGC
P9 (*lppA*-test-F)	TATTTGGCAGGGCTTCTCGA
P10 (*lppA*-test-F)	AATTGCCCTGCGTGTCTTTT

### Growth analysis of wild-type *H. parasuis*, *δlppa, and C-lppA* strains

Wild-type *H. parasuis* SH0165 and its derivatives *ΔlppA* and C-*lppA* were incubated overnight in TSB medium supplemented with NAD and serum, and then the cultures were diluted to an OD600 value of 1.0, before inoculation at a 1:1000 ratio into fresh medium. Fresh cultures were incubated at 37 °C on a shaker (180 rpm) for 4 h. The OD600 value of the cultures was detected with an Eppendorf Biospectrometer (Eppendorf, Hamburg, Germany) at 3 h intervals.

### Animal experiments and cell collection

The 30-day-old piglets were randomly divided into three groups and intraperitoneally injected with wild-type *H. parasuis* or Δ*lppA* strains at a dose of 1 × 10^9^ CFU. Piglets from the uninfected group were intraperitoneally injected with an equal volume of normal saline. Piglets treated with the wild-type *H. parasuis* revealed significant clinical symptoms including fever, inappetence, arthritis, breathing difficulty and cough at 4 h post-inoculation, the blood of piglets in different groups was collected for serum isolation and subsequent enzyme-linked immunosorbent assay (ELISA) analysis. All piglets were euthanized 4 h after injection. The primary PAMs of every piglet were collected from bronchoalveolar lavage fluid, as previously described [18], to analyse resistin expression using qRT-PCR. The primary PAECs of all piglets were separated from the porcine aorta to detect claudin-5 and occludin expression using qRT-PCR.

### Statistical analysis

Data are presented as mean ± standard error of the mean (SEM). All experiments were independently conducted at least three times, and statistical significance was determined using one-way analysis of variance followed by Dunnett’s post-test using Prism 8 (GraphPad Prism 9). *P*-values of<0.05 were considered significant and<0.01 were highly significant.

## Results

### Resistin induces the monolayer leakage of PAECs during *H. parasuis* infection

**Figure 1.**
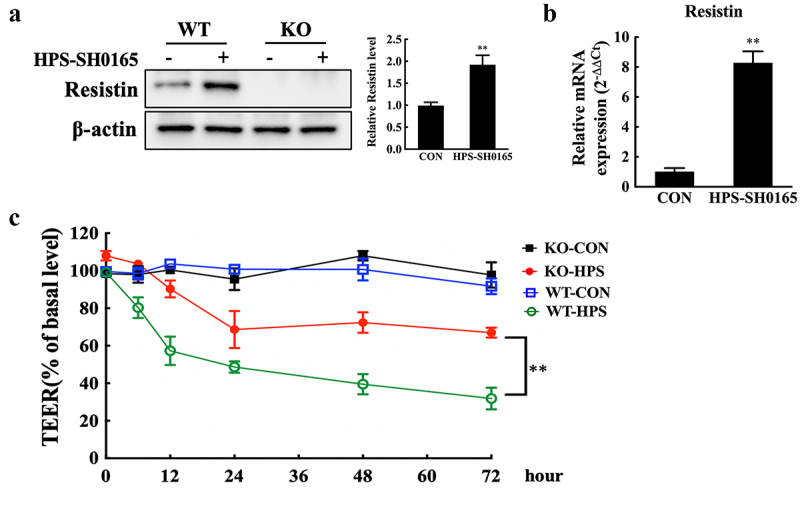
Resistin secreted by PAMs damages monolayer PAECs integrity. **(a, b)** *H. parasuis* (100 MOI) infected wild-type or resistin knockout PAMs. PAMs lysates were taken 2 h after infection. The resistin expression was determined using western blot analysis (A), and the mRNA *resistin* level was analysed using qRT-PCR (B). The antibodies against resistin and β-actin were diluted at 1/1000 and 1/50000, respectively. β-actin served as a loading control, and the relative resistin levels of wild-type cells were calculated with ImageJ software and normalized to β-actin. **p<0.01 compared with the uninfected wild-type group. **(c)** Wild-type or resistin knockout PAMs were co-cultured with PAECs. TEER of PAECs was measured at 0, 6, 12, 24, 48, and 2 h after PAMs were infected with *H. parasuis* (100 MOI). The TEER levels were displayed as a percentage of the TEER before treatment. The mean ± SEM (*n* = 3) is presented as a representative result of three independent tests. **p<0.01 compared with the wild-type group.

*H. parasuis* infection induced significant upregulation of resistin in PAMs (Figure 1(a,b) and S2A). We first generated a resistin-knockout PAM cell line using CRISPR/Cas9-mediated genome editing (Figure 1(a)) to investigate the role of resistin during *H. parasuis* infection, considering the potential effect of resistin on endothelial barrier integrity [7,8]. Wild-type or resistin-knockout PAMs were co-cultured with PAECs using a transwell system. Transendothelial resistance was measured after the PAMs were infected with *H. parasuis*. Knockout of resistin in PAMs significantly repaired the permeability of monolayer PAECs during *H. parasuis* infection compared to PAECs co-cultured with wild-type PAMs (Figure 1(c)). These results suggest that*H. parasuis*-induced resistin expression in PAMs may lead to monolayer leakage of PAECs.

### *H. parasuis*-induced resistin inhibits the expression of claudin-5 and occludin in PAECs

**Figure 2.**
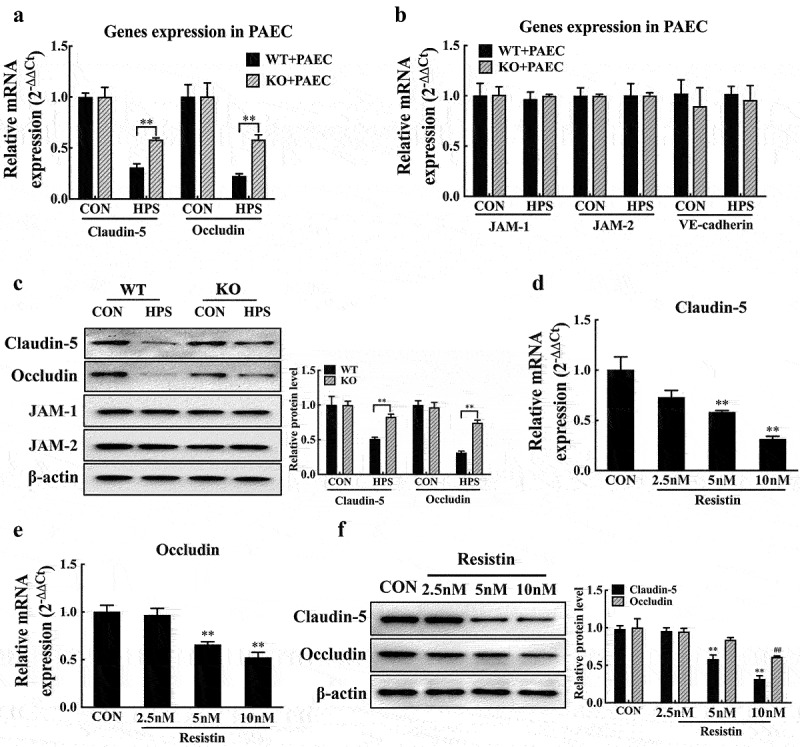
Resistin inhibits claudin-5 and occludin expression in PAECs. **(a, b, c)** PAECs were co-cultured with wild-type or resistin knockout PAMs, and the PAMs were infected by *H. parasuis* (100 MOI) for 2 h. mRNA levels of claudin-5, occludin, *JAM-1*, *JAM-2*, and VE-cadherin in PAECs were determined using qRT-PCR (a, b), and relative protein levels were detected using western blot analysis (c). **(d, e, f)** PAECs were stimulated or unstimulated with recombined resistin protein (2.5, 5, or 0 nM) for 2 h. Cells were lysed, mRNA levels of claudin-5 and occludin were analysed using qRT-PCR (d, e), and protein levels were analysed using western blot analysis (f). The mRNA level of each gene was standardized to GAPDH. The antibodies against claudin-5, occludin, JAM-1, JAM-2, and VE-cadherin were diluted at 1/1000, and the antibody against β-actin was diluted at 1/50000. β-actin served as a loading control, and the relative protein level was calculated with ImageJ software and normalized to β-actin. **p<0.01 compared with the wild-type group (a, b, c) or the untreated group (d, e, f). ^##^*p*<0.01 compared with the untreated group. Error bars represent the mean ± SEM (*n* = 3).

Tight and adherens junctions form the main connection between monolayer endothelial cells [11,12]; thus, we further investigated the expression levels of the major protein components of intercellular junctions in PAECs co-cultured with PAMs. The expression levels of claudin-5 and occludin in PAECs were markedly decreased after stimulation of PAMs co-cultured with *H. parasuis*, whereas resistin deletion in PAMs distinctly recovered claudin-5 and occludin expression levels in co-cultured PAECs, as shown inFigure 2(a) and S2C. However, the absence of resistin did not affect JAM-1, JAM-2, or VE-cadherin levels (Figure 2(b,c) [12]. These results suggest a potential role for resistin in regulating tight junctions of PAECs. A different dose of recombinant porcine resistin protein was used to stimulate PAECs to further confirm the function of resistin in the tight junctions and endothelial barrier. Resistin inhibited the expression of claudin-5 and occludin in a dose-dependent manner at both the mRNA and protein levels, as shown inFigure 2(d-f). These results indicate that *H. parasuis*-induced resistin in PAMs inhibits claudin-5 and occludin expression in PAECs, thereby destroying endothelial barrier integrity.

### Resistin modulates claudin-5 and occludin expression in PAECs by AMPK/mTOR signalling pathway

**Figure 3.**
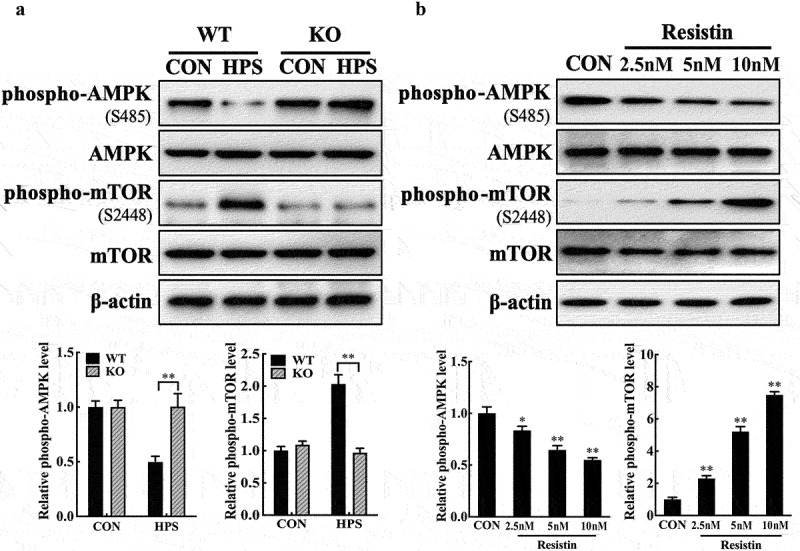
Resistin regulates AMPK/mTOR pathway activity in PAECs. **(a)** PAECs were co-cultured with wild-type or resistin knockout PAMs, and the PAMs were infected by *H. parasuis* (100 MOI) for 2 h. Lysates of PAECs were collected and subsequently analysed for the protein levels of phospho-AMPK (S458), AMPK, phospho-mTOR (S2448), and mTOR using western blot analysis. β-actin served as a loading control. **(b)** PAECs were stimulated or unstimulated with recombined resistin protein (2.5, 5, or 0 nM) for 2 h. The expression of related proteins was analysed the same as above. The antibodies against phospho-AMPK (S458), AMPK, phospho-mTOR (S2448), and mTOR were diluted at 1/1000, and the antibody against β-actin was diluted at 1/50000. β-actin served as a loading control, and the relative protein level was calculated with ImageJ software and normalized to β-actin. **p<0.01 compared with the wild-type group (a) or the untreated group (b). Error bars represent the mean ± SEM (*n* = 3).

We tested the activity of the AMPK/mTOR signalling pathway, which is a potential target pathway of resistin in cardiovascular diseases, to further determine the molecular mechanism of *H. parasuis*-induced resistin in regulating claudin-5 and occludin expressions in PAECs [23,24]. Stimulation of PAMs with *H. parasuis* reduced the activity of AMPK in co-cultured PAECs and induced the activity of mTOR, as shown inFigure 3(a) and S2B. Moreover, resistin deletion restored the phosphorylation of AMPK and mTOR in PAECs during *H. parasuis* infection. These results suggest a role for resistin in regulating AMPK/mTOR pathway activity. Consistently, treatment of PAECs with recombinant porcine resistin modulated AMPK and mTOR phosphorylation (Figure 3(b)). These results indicate that *H. parasuis*-induced resistin in PAMs regulates AMPK/mTOR pathway activity in PAECs.

We further investigated the effects of the AMPK/mTOR pathway on claudin-5 and occludin expression because resistin secreted by PAMs might promote *H. parasuis*-induced endothelial barrier defects by inhibiting claudin-5 and occludin production (Figure 2). AMPK activation and mTOR inhibition could mitigate claudin-5 and occludin expression suppression caused by resistin, as well as repair the monolayer permeability of PAECs stimulated with resistin, as shown inFigure 4. Therefore, resistin secreted by PAMs may regulate claudin-5 and occludin expression in PAECs through the AMPK/mTOR signalling pathway.

**Figure 4.**
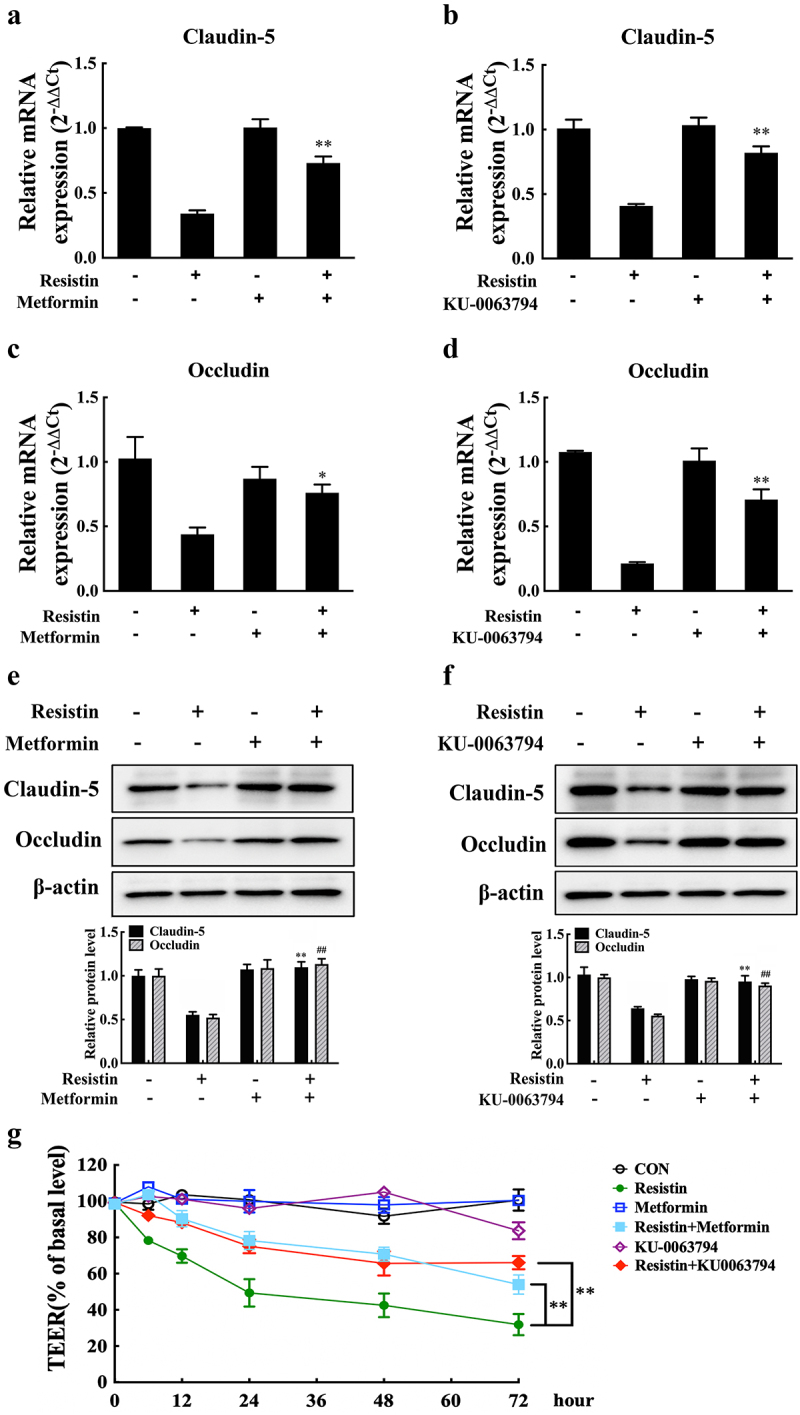
Resistin modulates claudin-5 and occludin expression through the AMPK/mTOR pathway. PAECs were treated or untreated with AMPK activator (Metformin) or mTOR inhibitor (KU-0063794) for 2 h before being stimulated or unstimulated with recombined resistin protein (2.5, 5, or 0 nM) for 2 h. mRNA levels of claudin-5 and occludin were analysed using qRT-PCR **(a-d)** and protein levels were analysed using western blot analysis **(e, f)**; the mRNA levels of each gene were standardized to GAPDH. The antibodies were diluted as previously described. β-actin served as a loading control, and the relative protein level was calculated with ImageJ software and normalized to β-actin. **p<0.01 and ^##^*p*<0.01 compared with the resistin-treated (without inhibitors) group. Error bars represent the mean ± SEM (*n* = 3). **(g)** TEER of PAECs was measured at 0, 6, 12, 24, 48, and 2 h after recombined resistin protein treatment (0 nM). The TEER levels were displayed as a percentage of the TEER before treatment. **p<0.01 compared with the untreated group. Error bars represented the mean ± SEM (*n* = 3).

### Resistin regulates AMPK/mTOR pathway activity and claudin-5 and occludin expression through the kinase LKB1

**Figure 5.**
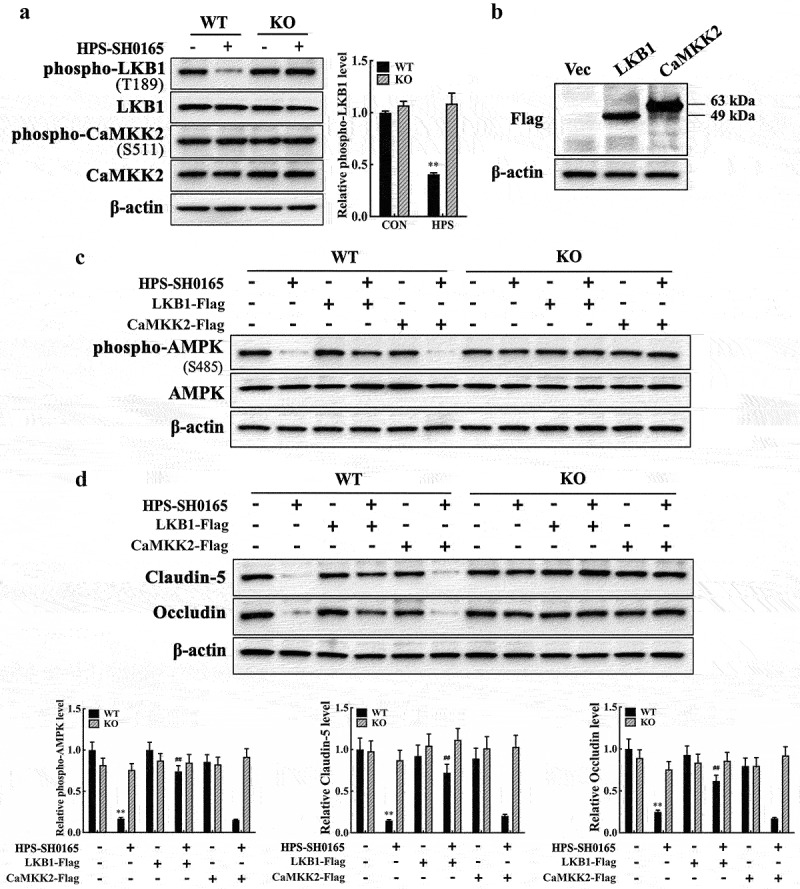
*H. parasuis*-induced resistin in PAMs modulates AMPK/mTOR pathway activity through LKB1 in PAECs. **(a)** PAECs were co-cultured with wild-type or resistin knockout PAMs, and the PAMs were infected by *H. parasuis* (100 MOI) for 2 h. Protein levels of phospho-LKB1 (T189), LKB1, phospho-CaMKK2 (S511), and CaMKK2 in PAECs were detected using western blot. **(b)** Western blot analysis of full-length LKB1 or CaMKK2 expression in PAECs. PAECs seeded on six-well plates were transfected with 2.5 μg/well plasmids pCAGGS-LKB1 or pCAGGS-CaMKK2 by 5 μg/well of lipofectamine 2000. Cells were collected to detect LKB1 and CaMKK2 expression levels using western blot analysis at 8 h post-transfection. The empty vector was used as a negative control. **(c, d)** pCAGGS-LKB1 or pCAGGS-CaMKK2 were transfected into PAECs, cells were co-cultured with wild-type or resistin knockout PAMs 4 h after transfection, and the PAMs were then infected by *H. parasuis* (100 MOI) for 2 h. Protein levels of phospho-AMPK (S458) and AMPK **(c)** or claudin-5 and occludin **(d)** in PAECs were determined using western blot analysis. β-actin served as a loading control. The antibodies against phospho-LKB1 (T189), LKB1, phospho-CaMKK2 (S511), and CaMKK2 were diluted at 1/1000, and the antibody against β-actin was diluted at 1/50000. β-actin served as a loading control, and the relative protein level was calculated with ImageJ software and normalized to β-actin. **p<0.01 compared with the uninfected group (a, c, d). ^##^*p*<0.01 compared with the HPS-infected group (c, d). Error bars represent the mean ± SEM (*n* = 3).

Previous studies have revealed that AMPK phosphorylation and subsequent signalling pathway activation require the kinase activity of LKB1 or CaMKK2 [25]. Hence, we first detected the phosphorylation levels of LKB1 and CaMKK2 in PAECs co-cultured with PAMs to further explore the signal cascade of the AMPK/mTOR pathway during resistin stimulation in PAECs. PAM stimulation with *H. parasuis* reduced LKB1 phosphorylation in co-cultured PAECs but did not influence CaMKK2 phosphorylation, as shown inFigure 5(a). We generated the eukaryotic expression plasmids pCAGGS-LKB1 and pCAGGS-CaMKK2 to further identify the effects of LKB1 and CaMKK2 (Figure 5(b)). LKB1 overexpression, rather than CaMKK2, in PAECs significantly recovered AMPK phosphorylation, as well as claudin-5 and occludin expression levels, which were repressed by *H. parasuis* stimulation in co-cultured PAMs (Figure 5(c,d)). Furthermore, resistin knockout in PAMs blocked the influence of *H. parasuis* on modulating AMPK pathway activity, as well as claudin-5 and occludin expression levels in PAECs (Figure 5(c,d)). These results indicate that LKB1, rather than CaMKK2, may be the upstream resistin sensor of the AMPK/mTOR pathway in PAECs.

We investigated claudin-5 and occludin expression levels, as well as the monolayer integrity of PAECs after LKB1 or CaMKK2 overexpression in the presence of resistin, to further identify the function of LKB1. LKB1 overexpression, rather than CaMKK2 overexpression, restored claudin-5 and occludin expression during resistin stimulation and recovered the TEER values of monolayer PAECs, which is consistent with the results above, as shown inFigure 6.

**Figure 6.**
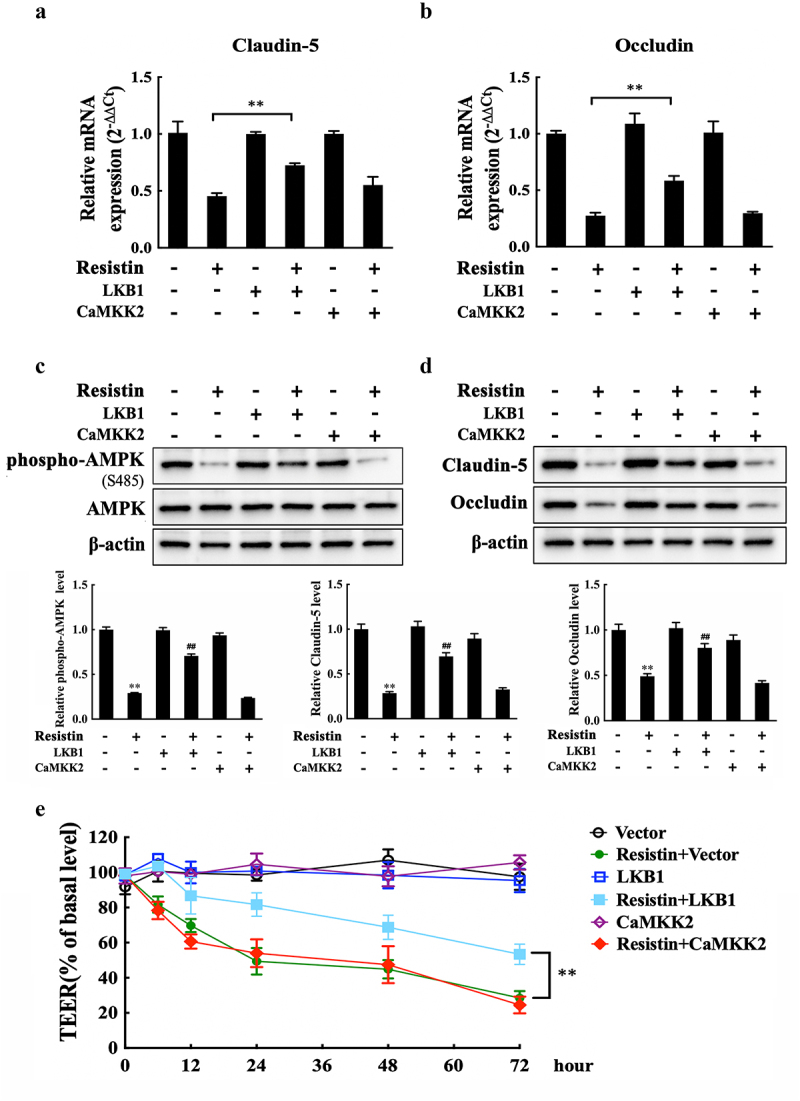
Resistin regulates monolayer PAECs integrity by the LKB1/AMPK/mTOR pathway. Plasmids pCAGGS-LKB1 or pCAGGS-CaMKK2 were transfected into PAECs. 4 h after transfection, PAECs were treated with recombined resistin protein (0 nM) for another 2 h, and cells were then collected for qRT-PCR analysis **(a, b)** or western blot analysis **(c, d)**. The empty vector was used as a negative control. The antibodies were diluted as previously described. β-actin served as a loading control, and the relative protein level was calculated with ImageJ software and normalized to β-actin. **p<0.01 compared with the untreated group, ^##^*p*<0.01 compared with the resistin-treated group. Error bars represent the mean ± SEM (*n* = 3). **(e)** TEER measurement of PAECs was taken at 0, 6, 12, 24, 48, and 2 h after recombined resistin protein treatment (0 nM). TEER levels were displayed as a percentage of the TEER before treatment. **p<0.01 compared with the negative control group. Error bars represented the mean ± SEM (*n* = 3).

### *LppA* gene of *H. parasuis* inhibits claudin-5 and occludin expression by promoting resistin expression

**Figure 7.**
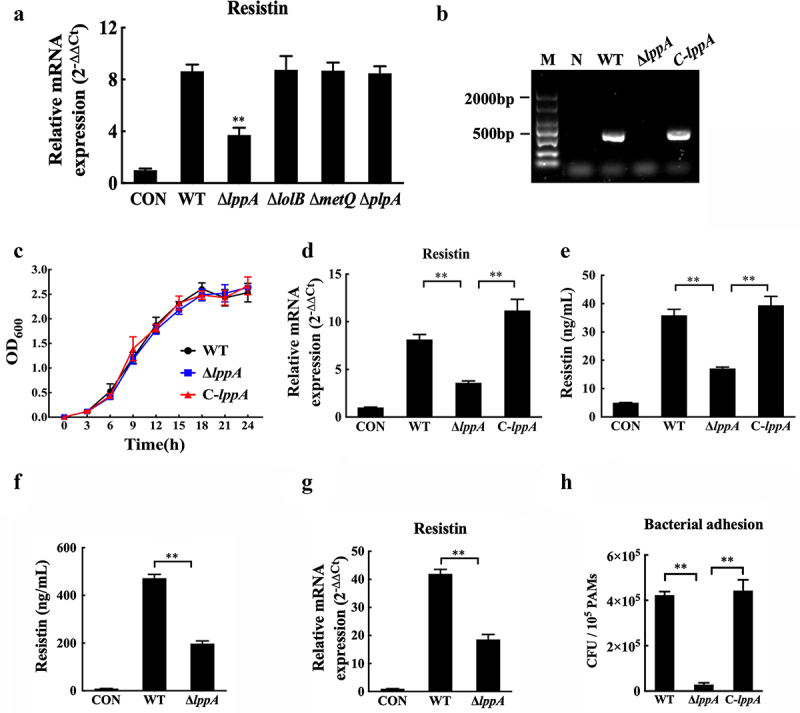
*H. parasuis* LppA induces resistin expression *in vitro* and *in vivo*. **(a)** PAMs were infected by wild-type SH0165, *δlppa* mutants, *δlolb* mutants, *δmetq* mutants, or *δplpa* mutants (100 MOI) for 2 h, and resistin expression in PAMs was determined using qRT-PCR. The mRNA levels of each gene were standardized to GAPDH. **p<0.01 compared with the wild-type SH0165 group. **(b)** PCR detection of the *lppA* gene deletion mutant and the complementation strain. Bacteria cells were collected to obtain total RNA, which was subsequently reverse-transcribed into cDNA. Primers P9 and P10 were used to detect *lppA* gene expression using PCR. WT: wild-type *H. parasuis*, NC: negative control, M: DL2000 marker. **(c)** the growth status of the wild-type SH0165, Δ*lppA* mutant, and C-*lppA* strains. Bacteria cells were cultured for 4 h, and OD600 values were measured every 3 h for each strain. **(d, e)** PAECs were co-cultured with wild-type PAMs, and PAMs were infected by wild-type SH0165, *δlppa* mutant, or C-*lppA* strains (100 MOI) for 2 h. The supernatant and lyses of these PAMs were then collected. The *resistin* expression in PAMs was analysed using qRT-PCR **(d)**. The mRNA levels of each gene were standardized to GAPDH. The supernatant of PAMs was collected for ELISA analysis **(e)**. **(f, g)** Resistin levels in serum **(e)** and primary porcine alveolar macrophages **(g)** were analysed using ELISA and qRT-PCR, respectively. **(h)** Adhesion to PAMs by wild-type SH0165, *δlppa* mutant, or C-*lppA* strains (100 MOI). **p<0.01. Error bars represent the mean ± SEM (*n* = 3).

The outer membrane lipoproteins of gram-negative bacteria have been demonstrated to play a vital role in promoting pathogen adhesion to host cells and in translocating virulence factors into host cells [26,27]. We generated the deletion mutants of the main outer membrane lipoprotein genes (*lppA, lolB*, *metQ*, and *plpA*) in *H. parasuis* to infected PAMs respectively, and the results revealed that the outer membrane lipoprotein gene *lppA*, rather than *lolB*, *metQ*, or *plpA*, in *H. parasuis* might participate in triggering resistin production in PAMs (Figure 7(a)). Thus, we constructed the *lppA* deletion mutant (*ΔlppA*) and the related complemented strain (C-*lppA*) for subsequent research. The *lppA* gene was found to exist in wild-type *H. parasuis* and the C-*lppA* strain but not in the deletion mutant, as shown inFigure 7(b).

Wild-type *H. parasuis*, *ΔlppA*, and C-*lppA* strains showed similar growth characteristics at 37 °C (Figure 7(c)), and the C-*lppA* strain recovered the induction of resistin production (Figure 7d,e). Moreover, the deletion of *lppA* significantly decreased resistin levels in porcine serum and primary PAMs induced by *H. parasuis* infection (Figure 7(f,g). We further explored the mechanism by which LppA regulates resistin expression in PAMs and found that its deletion markedly decreased *H. parasuis* adhesion to PAMs (Figure 7(h)). Furthermore, compared to that of wild-type *H. parasuis*, the culture supernatant of the *ΔlppA* strain induced a lower resistin expression level in PAMs (Fig. S3A). However, the recombinant LppA protein of *H. parasuis* did not increase resistin expression in PAMs (Fig. S3B). These results reveal that, in *H. parasuis*, LppA might participate in increasing resistin production by regulating bacterial adhesion to host cells and changing some bacterial factor production responsible for the induced resistin expression rather than directly inducing resistin production.

**Figure 8.**
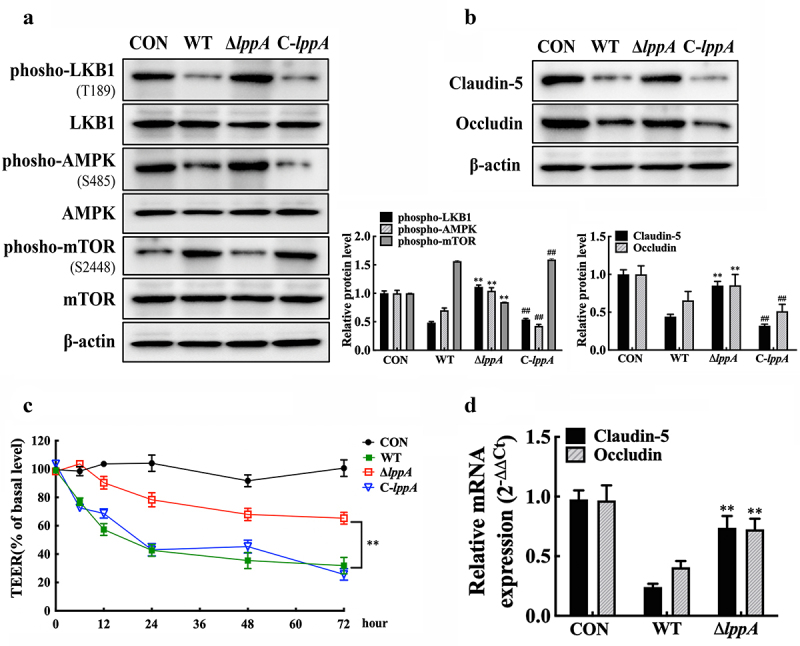
*H. parasuis* LppA reduces porcine endothelial cell integrity. **(a, b)** PAECs were co-cultured with wild-type PAMs, and PAMs were infected by wild-type SH0165, *δlppa* mutant, or C-*lppA* strains (100 MOI) for 2 h. Protein expression levels of phospho-LKB1 (T189), LKB1, phospho-AMPK (S458), AMPK, phospho-mTOR (S2448), and mTOR in PAECs **(a)**, as well as the claudin-5 and occludin **(b)**, were determined using western blot analysis. The antibodies were diluted as described above. β-actin served as a loading control, and the relative protein level was calculated with ImageJ software and normalized to β-actin. **p<0.01 compared with the untreated group, ^##^*p*<0.01 compared with the wild type *H. parasuis*-infected group. **(c)** TEER of PAECs was measured at 0, 6, 12, 24, 48, and 2 h after infecting PAMs by wild-type SH0165, *δlppa* mutant, or C-*lppA* strains (100 MOI). TEER levels were displayed as a percentage of the TEER before treatment. **p<0.01 compared with the wild-type SH0165 group. **(d)** claudin-5 and occludin expression in primary PAECs was analysed using qRT-PCR. **p<0.01 compared with the wild-type SH0165 group. Error bars represent the mean ± SEM (*n* = 3).

Wild-type *H. parasuis*, *ΔlppA*, and C-*lppA* were used to infect PAMs in the transwell cell culture system to further explore the role of the LppA in *H. parasuis*-induced endothelial integrity damage. The co-cultured PAECs were collected to examine the activity of the LKB1/AMPK/mTOR pathway and monolayer integrity. *lppA* deletion markedly recovered the phosphorylation levels of LKB1, AMPK, and mTOR (Figure 8(a)), thereby restoring claudin-5 and occludin expression (Figure 8(b)) and concurrently decreasing the injury of monolayer PAECs integrity (Figure 8(c)), compared with wild-type *H. parasuis*. Moreover, the *in vivo* experiment revealed a significant increase in claudin-5 and occludin expression levels in the primary PAECs of piglets treated with the *ΔlppA* strain compared with the wild-type *H. parasuis* treated group (Figure 8(d)). These results reveal that LppA in *H. parasuis* promote endothelial barrier dysfunction by inducing resistin expression in PAMs.

## Discussion

Resistin, a multifunctional peptide hormone, is associated with various pathological processes, including obesity, type 2 diabetes, and cancer cell proliferation [28,29]. Furthermore, resistin plays a crucial role in regulating VE cell function. A previous study demonstrated that resistin stimulates endothelin 1 (ET-1) and vascular endothelial growth factor receptor (VEGFR-1 and VEGFR) production in HCAEC to promote angiogenesis *in vitro* as well as endothelial cell proliferation and migration [30,31]. Additionally, resistin in rats was found to control the release of plasminogen activator inhibitor 1 (PAI-1), von Willebrand Factor (vWF), and ET-1 via the PI3K pathway, resulting in rat endothelial cell dysfunction [32]. These results suggest an important role for resistin in modulating endothelial cell function; however, few studies have focused on the role of resistin in affecting the permeability of monolayer endothelial cells and regulating endothelial integrity, particularly in infectious diseases. Resistin reduces the expression of tight junction – associated proteins, including occludin and ZO-1, in HCAEC and enhances the permeability of monolayer HCAEC in a non-infectious model [8]. However, the role of resistin in pathogen infections remains unclear. In this study, we constructed an *in vitro* cell co-culture system to mimic the *in vivo* infection process of *H. parasuis* and investigated the effects of resistin released by PAMs on endothelial integrity. Our results indicate that resistin secreted by PAMs reduces claudin-5 and occludin expression in co-cultured PAECs and increases the permeability of monolayer PAECs, implying that resistin plays an important role in maintaining endothelial integrity during pathogen infection.

In a previous study, we confirmed that *H. parasuis* induces epithelial-mesenchymal transition (EMT) in porcine epithelial cells by activating the Wnt/β-catenin pathway, thereby damaging the epithelial barrier [33]. Furthermore, resistin accelerates the EMT process in ovarian cancer cells by decreasing the expression of the epithelial cell marker E-cadherin and boosting the expression of the mesenchymal cell markers ZEB1 and Vimentin, thereby encouraging cancer cells to migrate [29,34]. However, resistin did not trigger the process of EMT in PAECs, as VE-cadherin expression levels, which are endothelial cell marker genes, were not significantly altered in the absence of resistin. Similarly, Jamaluddin et al. revealed that resistin does not affect VE-cadherin expression levels in HCAECs [8]. Therefore, we speculate that resistin may trigger different mechanisms to alter the integrity of the epithelial and endothelial barriers during *H. parasuis* infection, but identifying these mechanisms requires further study.

The present study revealed that the outer membrane lipoprotein LppA of the *H. parasuis* SH0165 strain participates in regulating resistin expression in PAMs, thereby influencing the permeability of monolayer PAECs. The specific function of the *lppA* gene in *H. parasuis* is yet to be determined, but studies focusing on the outer membrane proteins of gram-negative bacteria have defined the role of outer membrane lipoproteins in cell wall synthesis, diverse secretion systems, and antibiotic efflux pumps in bacteria [26]. Additionally, lipoproteins play essential roles in host cell adhesion and virulence factor translocation into host cells, thereby influencing the inflammatory process [26,27]. Consistent with these studies, our work suggests that *lppA* deletion decreases *H. parasuis* adhesion to PAMs as well as resistin expression in PAMs induced by *H. parasuis*. Therefore, LppA in *H. parasuis* might enhance bacterial adherence to PAMs and induce bacterial factor production responsible for resistin expression. This would then trigger the inflammatory response by stimulating the release of pro-inflammatory cytokines, such as resistin, and promoting inflammatory damage in host cells. However, the detailed pathogenic mechanism of LppA in *H. parasuis* requires further investigation.

**Figure 9.**
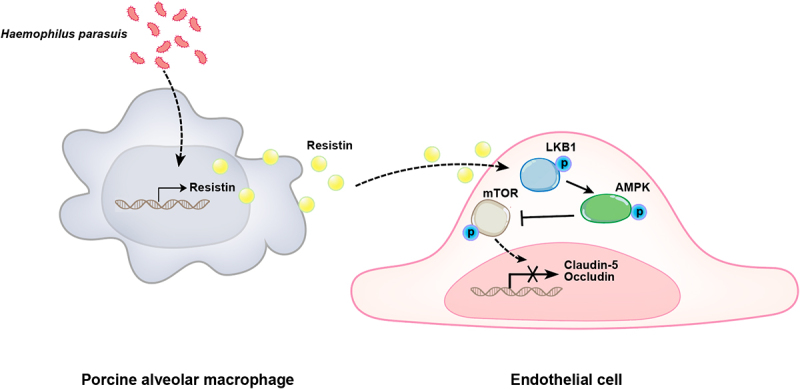
Schematic diagram of the potential cell signalling mechanisms responsible for resistin-induced endothelial cell dysfunction during *H. parasuis* infection. *H. parasuis* stimulated PAMs to secret resistin, which inhibited claudin-5 and occludin expression in PAECs through the LKB1/AMPK/mTOR pathway, thereby damaging the endothelial cell monolayer integrity.

In conclusion, we discovered that the LppA of *H. parasuis* stimulates PAMs to secrete resistin, which inhibits tight junction protein expression, including claudin-5 and occludin, in PAECs via the LKB1/AMPK/mTOR pathway. This then damages endothelial cell monolayer integrity (Figure 9). Therefore, the present study elucidates the potential molecular mechanisms of resistin-induced endothelial barrier dysfunction during *H. parasuis* infection and provides fresh insight into the pathogenesis of Glässer’s disease, which is characterized by exudative inflammation.

## Supplementary Material

Supplemental Material
