# Supplementary material for: Resistin secreted by porcine alveolar macrophages leads to endothelial cell dysfunction during Haemophilus parasuis infection
Source: Virulence. 2023 Feb 8;14(1):2171636. doi: 10.1080/21505594.2023.2171636 (PMC9928480; doi:10.1080/21505594.2023.2171636)
Supplement: Supplemental Material [file KVIR_A_2171636_SM6622.docx]

**Supplemental Figures**

# Resistin secreted by porcine alveolar macrophages leads to endothelial cell dysfunction during *Haemophilus parasuis* infection

Kexin Hua^1, 2, 3^, Tingting Li^4^, Yanling He^1, 2, 3^, Aohan Guan^1, 2, 3^, Liying Chen^1, 2, 3^, Yuan Gao^1, 2, 3^, Qianshuan Xu^1, 2, 3^, Haoyu Wang^1, 2, 3^, Rui Luo^1, 2, 3^, Ling Zhao^1, 2, 3^, Hui Jin^1, 2, 3*^

^1^State Key Laboratory of Agricultural Microbiology, Huazhong Agricultural University, China, ^2^College of Veterinery Medicine, Huazhong Agricultural University, China, ^3^Hubei Provincial Key Laboratory of Preventive Veterinary Medicine, Huazhong Agricultural University, China, ^4^Hubei Animal Disease Prevention and Control Center, Wuhan, China

*Corresponding authors Address: State Key Laboratory of Agricultural Microbiology, College of Veterinary Medicine, Huazhong Agricultural University, No.1 Shizishan Road, Wuhan 430070, People’s Republic of China.

E-mail: [jinhui@mail.hzau.edu.cn](mailto:jinhui@mail.hzau.edu.cn) (H. Jin)

**Running title :** HPS damages endothelial cell integrity via resistin


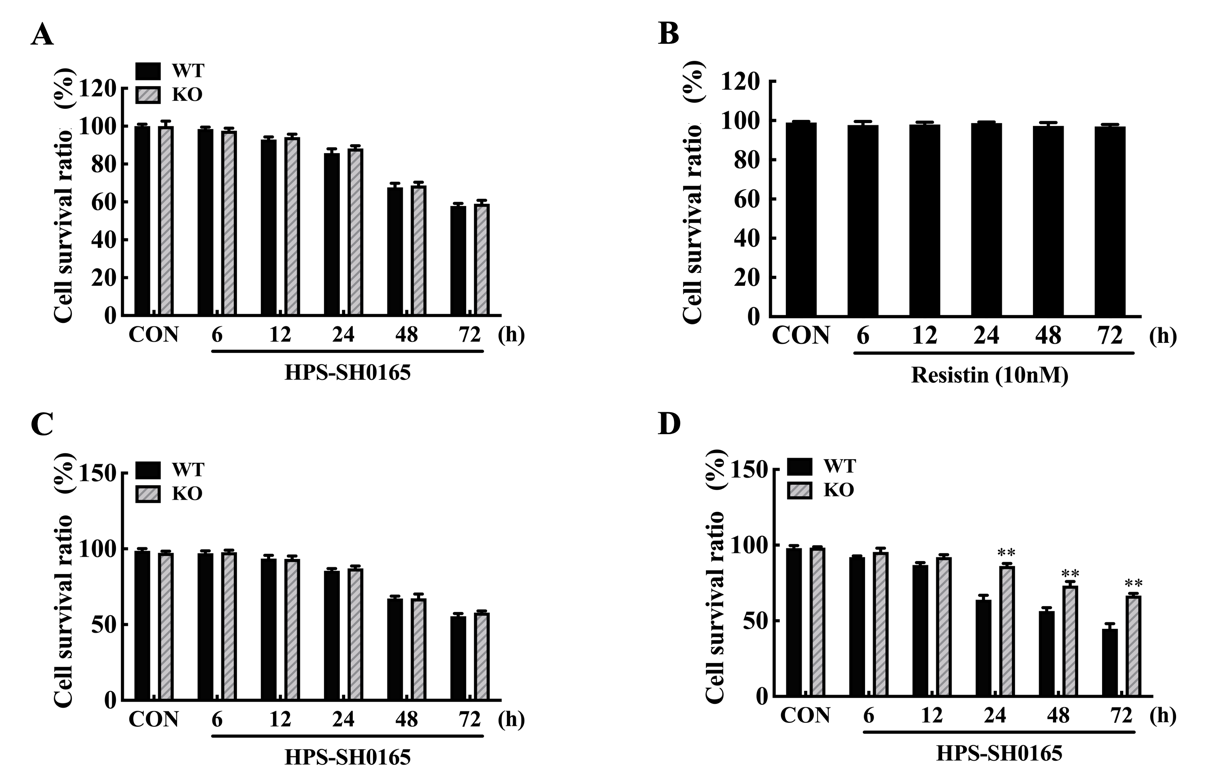


**Supplemental Figure S1 :** The cell survival ratio of PAMs and PAECs. **(A)** Wild type or resistin knockout PAMs were infected with *H.* *parasuis* (100 MOI) for 6 h, 12 h, 24 h, 48 h, and 72 h. **(B)** PAECs were treated by Resistin (10nM) for 6 h, 12 h, 24 h, 48 h, and 72 h. **(C, D)** Wild type or resistin knockout PAMs (C) and PAECs (D) were co-cultured in the transwell system. PAMs were infected with *H. parasuis* (100 MOI) for 6 h, 12 h, 24 h, 48 h, and 72 h. Cells were stained by trypan blue and the survival cells (unstained) and dead cells (stained) were counted separately in the hemacytometer. ***p* < 0.01 compared with the wild-type group. Error bars represented the mean +/- SEM (n = 3).


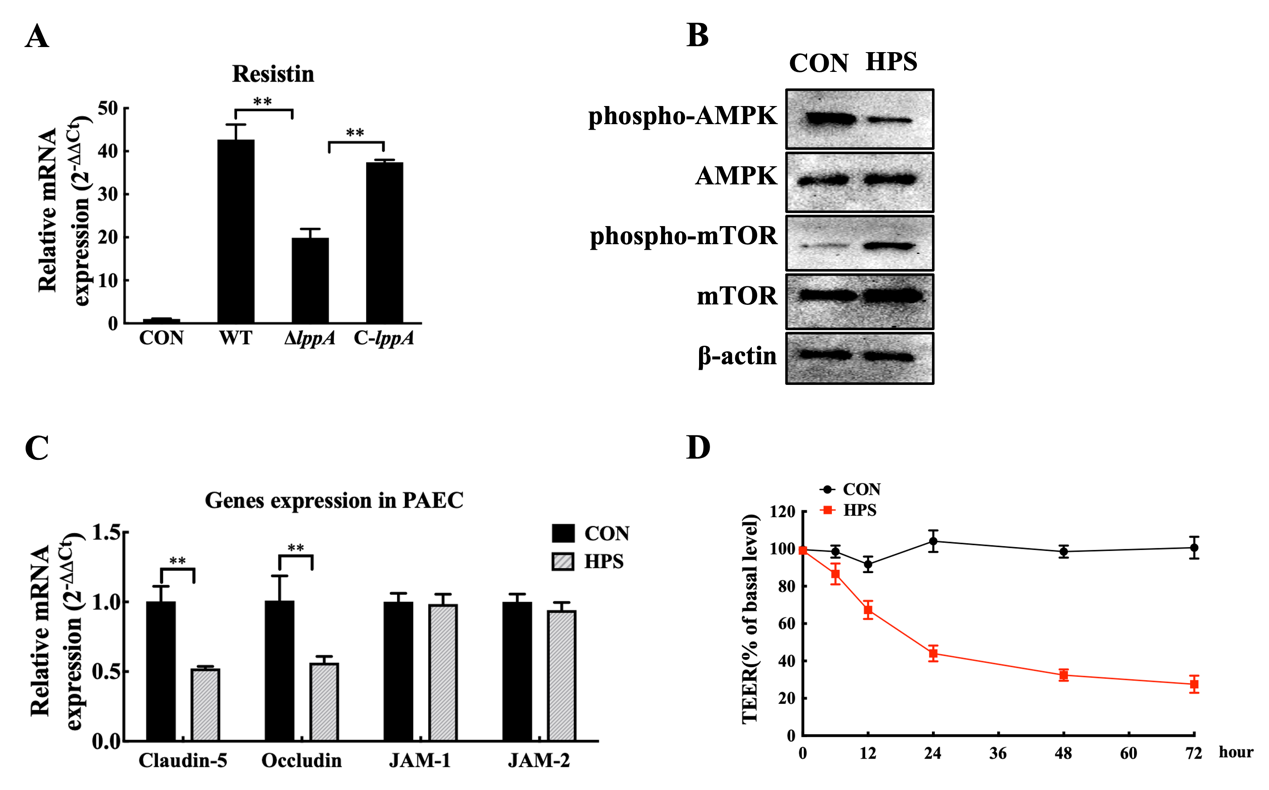


**Supplemental Figure S2 :** *H. parasuis* induced the expression of resistin in primary porcine alveolar macrophages (pPAMs), inhibited claudin-5 and occludin expression in co-cultured PAECs and increased the permeability of monolayer PAECs. **(A)** pPAMs were infected by wild-type SH0165, Δ*lppA* mutant, or C-*lppA* strains (100 MOI) for 12h and resistin expression in pPAMs was determined by qRT-PCR. The mRNA level of resistin was standardized to GAPDH. **(B-D)** PAECs were co-cultured with pPAMs, and pPAMs were infected by wild type SH0165 for 12h. The protein levels of phospho-AMPK (S458), AMPK, phospho-mTOR (S2448), and mTOR in PAECs were determined by western blot (B). The claudin-5 and occludin expression in PAECs was analyzed by qRT-PCR and the mRNA levels of each gene were standardized to GAPDH (C). TEER of PAECs was measured at 0 h, 6 h, 12 h, 24 h, 48 h, and 72 h after infected pPAMs with *H. parasuis* (100 MOI). The TEER levels were displayed as a percentage of the TEER before treatment (D). ***p* < 0.01. Error bars represented the mean +/- SEM (n = 3).


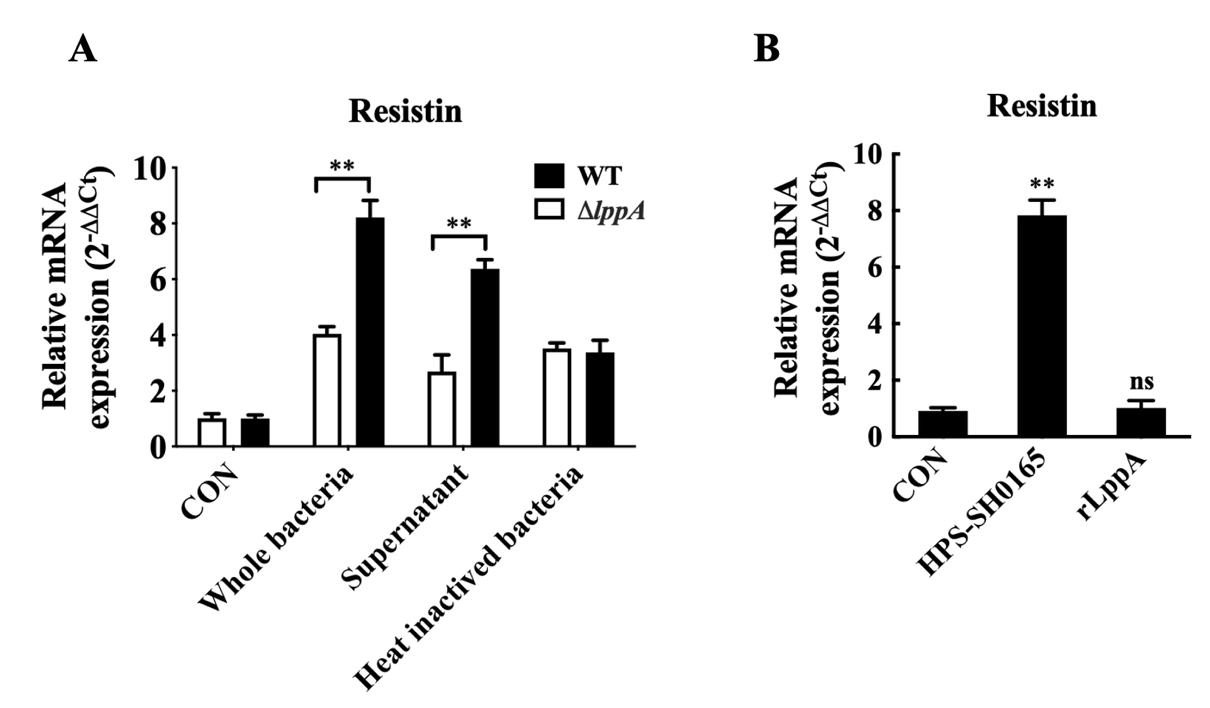


**Supplemental Figure S3 :** The effect of the LppA in *H. parasuis* on resistin expression in PAMs. **(A)** The LppA in *H. parasuis* promoted the resistin expression in PAMs induced by *H. parasuis* culture supernatant. PAMs were treated with the wild-type or the *lppA* gene deleted whole bacteria (100 MOI), the heat inactived bacteria (100 MOI), or the culture supernatant of bacteria for 12 h. Resistin expression in PAMs was determined by qRT-PCR. The mRNA level of resistin was standardized to GAPDH. ***p* < 0.01. Error bars represented the mean +/- SEM (n = 3). **(B)** The recombinant LppA (rLppA) of *H. parasuis* did not induced the expression of resistin in PAMs. PAMs were infected with *H. parasuis* (100 MOI) for 12 h, or treated with rLppA (10μM) for 12h. Resistin expression in PAMs was determined by qRT-PCR. The mRNA level of resistin was standardized to GAPDH. ***p* < 0.01, ns : no significance compared with the untreated group. Error bars represented the mean +/- SEM (n = 3).
